# Impact of contrast enhancement boost and super-resolution deep learning reconstruction on pediatric congenital heart disease CTA scans: ultra-low contrast dose

**DOI:** 10.1186/s12880-025-02015-2

**Published:** 2025-11-18

**Authors:** Xinyan Zhou, Junyi Li, Tengfei Ke, Daoqiang Xiong, Fei Liu, Na Tan, Xirui Duan, Xiaolan Du, Feifei Zhou, Wan Shen, Rong Qian, Guochen Li, Guangrong Zheng, Lei Tang, Chengde Liao

**Affiliations:** 1 Present Address: Department of Radiology, Kunming Yan’an Hospital (Yan’an Hospital Affiliated to Kunming Medical University), Kunming, China; 2 Key Laboratory of Cardiovascular Disease of Yunnan Province, Kunming, China; 3 The First Affiliated Hospital of Kunming Medical University, Kunming, China; 4 Department of Radiology, Yunnan Cancer Hospital (The Third Affiliated Hospital of Kunming Medical University), Kunming, China; 5 Present Address: School of Media and Design Arts, Yunnan University of Finance and Economics, Kunming, China

**Keywords:** Low contrast dose, Pediatric, Cardiac computed tomography angiography, Congenital heart disease, Super-resolution deep learning reconstruction

## Abstract

**Objective:**

To evaluate the feasibility of using contrast enhancement boost (CE-Boost) combined with super-resolution deep learning reconstruction (SR-DLR) to reduce contrast agent dosage in pediatric patients with congenital heart disease (CHD).

**Methods:**

A total of 72 pediatric CHD patients were divided into the low-contrast-dose group (CE-Boost group, injection of 0.5 mL per kilogram of body weight, *n* = 36) or the standard-scan group (CE-CT group, 1.5 mL per kilogram of body weight, *n* = 36), both groups undergoing imaging with SR-DLR. The two imaging protocols were compared based on radiation dose, objective image quality, subjective evaluation, and diagnostic accuracy. To quantitatively evaluate image quality, CT attenuation (HU) and standard deviation (SD) values were measured within ROIs at the four-chamber plane for cardiac chambers; an anonymized dataset was assessed using a double-blind methodology by two independent readers blinded to the clinical information and prior diagnoses of the pediatric patients.

**Results:**

CE-Boost combined with SR-DLR significantly reduced contrast agent usage (62.3% reduction compared to CE-CT, *P* < 0.001) while maintaining image quality comparable to the conventional contrast protocol (*P* > 0.05). There was no significant difference in radiation dose parameters, including dose-length product (DLP, mGy·cm) and volume-weighted CT dose index (CTDIvol, mGy) (all *P* > 0.1), while the effective dose (ED) in the CE-Boost group was slightly lower but not significant (0.36 vs. 0.43 mSv, *P* = 0.078). Additionally, the CE-Boost group’s image quality metrics (CT values, SNR, CNR) remained stable, with no significant difference in subjective scores (*P* = 0.660).

**Conclusion:**

CE-Boost combined with SR-DLR enables a significant reduction in contrast agent usage in pediatric CHD imaging while maintaining comparable image quality to conventional contrast protocols and optimizing SNR and CNR. This approach ensures diagnostic readability while minimizing contrast exposure, highlighting its feasibility and clinical value in pediatric CHD imaging.

**Supplementary Information:**

The online version contains supplementary material available at 10.1186/s12880-025-02015-2.

## TOC Summary

This study evaluates the feasibility of CE-Boost combined with super-resolution deep learning reconstruction (SR-DLR) in reducing contrast agent usage for pediatric congenital heart disease (CHD) imaging. Compared to conventional contrast-enhanced CT, this approach achieved a 62.3% reduction in contrast dose (*P* < 0.001) while maintaining comparable image quality (*P* > 0.05). Radiation dose parameters showed no significant differences (*P* > 0.1). Image quality metrics (CT values, SNR, CNR) remained stable, ensuring diagnostic readability with minimized contrast exposure. These findings highlight the clinical potential of CE-Boost with SR-DLR in low-contrast-dose pediatric CHD imaging.

## Introduction

Congenital heart disease (CHD) is one of the most significant cardiac conditions in pediatrics and a leading cause of morbidity and mortality in neonates, affecting approximately 1% of live births worldwide [1]. CHD is characterized by structural abnormalities of the heart present at birth, affecting the heart’s function and blood circulation; thus, imaging plays a crucial role in diagnosing, classifying, and managing CHD. Advances in imaging techniques have significantly improved the detection and understanding of complex cardiac anomalies [2]. Key imaging modalities in CHD include echocardiography (ECHO), cardiac MRI (CMR), and computed tomography angiography (CTA). Yet, these modalities have certain limitations, such as limited acoustic windows in older children or post-surgical patients and operator-dependent requirements when using ECHO [3], time-consuming and contraindication for patients with certain metallic implants when using CMR [4], and intravenous administration of iodine-based contrast agents, which may pose long-term health risks to pediatric patients when CTA is applied [5]. Regarding CTA, studies have indicated that using iodinated contrast media is associated with increased radiation exposure to target organs [6, 7]. Yet, compared with adults, pediatric patients are considered more sensitive to ionizing radiation, with a sensitivity approximately ten times higher [8]. Additionally, infants are at greater risk of contrast agent-related adverse effects, including carcinogenesis and acute kidney injury [9, 10]. Thus, searching for methods to reduce agent dosage in pediatric CHD patients is essential.

Recently, the contrast enhancement boost (CE-Boost; Canon Medical Systems) post-processing technique has enabled the generation of subtraction images using a novel subtraction algorithm, which incorporates denoising processes to overlay these subtraction images onto the original images. CE-Boost aims to enhance the visibility of vascular structures in CTA imaging without necessitating additional contrast media or changes to scanning protocols [11]. However, one concern with post-processing techniques like CE-Boost is the potential increase in image noise, particularly in areas with low iodine concentration (e.g., small vessels or regions with poor contrast enhancement). Super-resolution deep learning reconstruction (SR-DLR) technology, which integrates deep convolutional neural networks into the image reconstruction process, has been suggested as an effective method to reduce image noise while preserving contrast in CE-Boost-enhanced images [12–14], and to significantly improve spatial resolution, noise texture, and lesion detectability [15, 16].

This study aims to explore the feasibility of combining SR-DLR with CE-Boost in low-contrast-dose CTA imaging for pediatric patients with CHD. We hypothesized that this combination could reduce the required contrast agent dose and maintain or enhance image quality while mitigating the potential risks associated with radiation and contrast agents. The ultimate goal of this study is to provide a safer, more effective imaging modality with clinical diagnostic value for pediatric CHD patients.

## Materials and methods

### Patients

This prospective study included data from 72 pediatric patients with CHD. The study adhered to the Declaration of Helsinki and was approved by the Medical Ethics Committee of Yan’an Hospital, Kunming City (Approval No.: 2024-006-01). Informed consent was obtained from all guardians of the enrolled children in compliance with the 1975 Declaration of Helsinki.

**Fig. 1:**
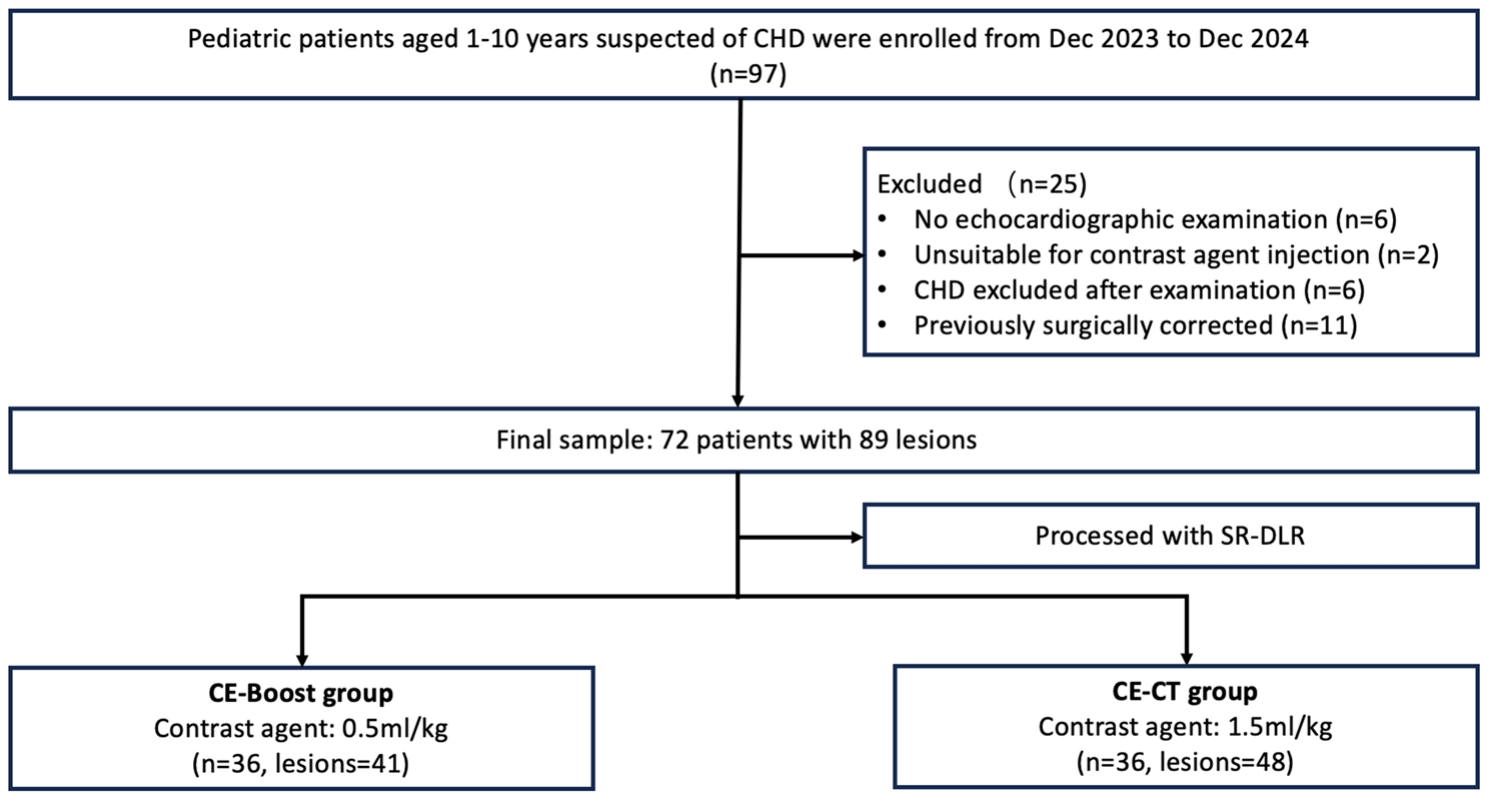
Flowchart of the patient enrollment process. CHD: congenital heart disease; SR-DLR: super-resolution deep learning reconstruction

In this single-center study, pediatric patients admitted to Yan’an Hospital Affiliated to Kunming Medical University for echocardiography-confirmed congenital heart defects and/or further surgical planning from December 2023 to December 2024 were assessed. Inclusion criteria were: children under 10 years of age with CHD and a chest-length (anteroposterior diameter) < 16 cm, ensuring full chest coverage with wide-detector CT; availability of comprehensive cardiac imaging, including CTA and echocardiography. CT indications for each participant were determined during weekly multidisciplinary team meetings involving pediatric cardiac surgeons, pediatric cardiologists, and qualified cardiovascular radiologists. Exclusion criteria included: (a) absence of echocardiographic examination, (b) unsuitable for contrast agent injection, (c) excluded from CHD diagnosis after examination, and (d) previously surgically corrected cases (Fig. 1).

Participants were randomly assigned to the low-contrast-dose group (CE-Boost group) or the standard-scan group (CE-CT group), with both groups undergoing imaging with SR-DLR. Patient characteristics are summarized in Table 1. Clinical data were extracted from the hospital’s electronic medical records.

**Table 1:** Patient characteristics

Characteristic	CE-CT Group (*n* = 36)	CE-Boost Group (*n* = 36)	*P*-value
Age (month)	69.42 ± 17.69	77.03 ± 24.29	0.157
Sex			0.808
Male	14(39)	13(36)	
Female	22(61)	23(64)	
Height (cm)	107.69 ± 15.87	114.75 ± 13.21	0.066
Weight (kg)	17.55 ± 5.05	19.89 ± 6.78	0.118
BMI (kg/m2)	14.88 ± 1.41	14.73 ± 2.19	0.753
Mean heart rate (/min)	104.42 ± 15.42	98.97 ± 12.66	0.150
Lesions	*n* = 48	*n* = 41	
ASD	29(60.4)	28(68.3)	
VSD	11(22.9)	7(17.1)	
PDA	5(10.4)	3(7.3)	
SAM	0	1(2.4)	
AOCA	1(2.1)	0	
PAPVC	1(2.1)	0	
DAA	1(2.1)	0	
PAVSD	0	2(4.9)	

Data are presented as numbers (percentages) or means ± standard deviations, with ranges in parenthe­ses. BMI: body mass index; ASD: atrial septal defect; VSD: ventricular septal defect; SAM: systolic anterior motion; AOCA: anomalous origin of the coronary artery; PAPVC: partial anomalous pulmonary venous connection; DAA: double aortic arch; PAVSD: partial atrioventricular septal defect

### Positioning for CT scanning and image reconstruction

Both the CE-Boost and CE-CT groups received the same contrast agent, iopamidol (370 mgI/mL, CTTQ), administered through a 22G × 1.00 in (0.9 × 25 mm) peripheral intravenous catheter placed in the right antecubital vein, using a dual-head power injector; the two groups differed only in the weight-to-contrast agent ratio. In the CE-Boost group, an iodine-based contrast agent was administered intravenously at 0.5 mL per kilogram of body weight, whereas in the CE-CT group, it was manually adjusted to 1.5 mL per kilogram of body weight. Scans were performed during free breathing in both groups. After contrast agent administration, an equivalent volume of normal saline (sodium chloride, 0.9%) was given at a rate of 1.5 mL per kilogram of body weight, with a 2-second delay before scan commencement. The injection protocol, including injection rate and three-phase scheme, was performed according to pediatric CTA guidelines [17, 18]. The injection rate of the contrast agent ranged from 0.3 to 2.0 mL/s.

A region of interest (ROI) was placed on the descending aorta with a triggering threshold set at 120 HU. All patients underwent imaging with a 16-cm wide-detector 320-row CT scanner (Aquilion ONE GENESIS, Canon Medical) using a prospective ECG-gated scanning mode. Scanning parameters are summarized in Table 2. The mean heart rate and its range for each patient were recorded.

**Table 2:** Scanning parameters and contrast medium for CTA

	CE-CT group	CE-boost group		*P*-value
Contrast medium parameters				
Scan mode	Volume scanning			
Tube voltage (kV)	80			
Tube current (mAs)	11–55			
Scanning and collimation (mm)	320 × 0.5			
Scan length (cm)	16			
ECG-gating technique	Prospective ECG-gating			
R-R interval	35–55%			
Reconstruction parameters				
Rotation time (S)	0.275			
Slice interval (mm)	0.5			
Slice thickness (mm)	0.5			
Scan FOV (mm)	220 mm			
Reconstruction FOV (mm)	220 mm			
Reconstruction kernel	Fc15			
Reconstruction algorithm	SR-DLR			
Radiation dose, Contrast Agent Dose				
Contrast volume (ml)	26.33 ± 7.57		9.94 ± 3.39	< 0.001
Injection rate (ml/s)	1.46 ± 0.39		1.59 ± 0.48	0.251
DLP (mGy·cm)	20.05 (15.88 − 27.83)		19.30 (14.58–21.40)	0.108
CTDIvol (mGy)	1.25 (0.99–1.74)		1.21 (0.91–1.34)	0.108
ED (mSv)	0.43 (0.30–0.59)		0.36 (0.34–0.40)	0.078

Data are presented as mean ± standard deviation or median (interquartile range). SR-DLR: super-resolution deep learning reconstruction; DLP: dose-length product; CTDIvol: the volume-weighted CT dose index; ED: effective dose

Raw data in both groups were reconstructed using a super-resolution deep learning reconstruction (SR-DLR; PIQE, Canon Medical Systems) to ensure a uniform reconstruction method. To generate CE-Boost images, noncontrast (precontrast) and arterial-phase CTA datasets covering the same anatomic volume were acquired. After nonrigid registration, the noncontrast image was subtracted from the CTA to derive an iodine map (removing background soft-tissue attenuation while retaining iodine signal), which was then fused with the original arterial-phase CTA and processed with automated denoising to produce contrast-enhanced CE-Boost images. All cases in the CE-Boost group followed this workflow. A representative arterial-phase CE-Boost image is shown in Fig. 5.

Multiplanar reconstructions (MPR) were performed on a clinical PACS workstation (Centricity 4.1). These were used to evaluate intracardiac structures, peripheral great vessels, and airways. Images from CE-Boost group patients were processed using the CE-Boost technique. Images in both groups were reconstructed using the SR-DLR algorithm.

### Radiation exposure

The CT console automatically recorded the dose-length product (DLP, mGy·cm) and volume-weighted CT dose index (CTDIvol, mGy). The effective dose (ED, mSv) was calculated following the European Guidelines on Quality Criteria for Computed Tomography [19], using age-specific conversion factors: 0.039 (< 4 months), 0.026 (4 months–1 year), 0.018 (1–5 years), and 0.013 (5–10 years) [20].

### Quantitative assessment of imaging

#### Objective evaluation

To quantitatively evaluate image quality, CT attenuation (HU) and standard deviation (SD) values were measured within ROIs at the four-chamber plane for cardiac chambers, the aorta (AO) at the level of the tracheal bifurcation, the pulmonary trunk (PT), and the erector spinae muscle. Care was taken to minimize artifacts from contrast agents when placing ROIs. Each ROI measurement was repeated three times at each location, and the average value was calculated to ensure data consistency. Signal-to-noise ratio (SNR) was defined as the mean CT attenuation of the vessel ROI divided by image noise, and contrast-to-noise ratio (CNR) as the difference between vessel attenuation and that of the erector spinae muscle divided by image noise, with image noise defined as the SD of the erector spinae ROI [21]. All measurements were performed on 0.5-mm reconstructions.

#### Subjective evaluation

An anonymized dataset was assessed using a double-blind methodology by two independent readers (both radiologists with over ten years of experience in cardiovascular imaging). The readers were blinded to the clinical information and prior diagnoses of the pediatric patients. Subjective image quality was rated on a 5-point scale (5 = excellent/absent, 4 = good/minimal, 3 = moderate, 2 = limited/substantial, 1 = poor/massive) [15]. The score reflected an overall impression of image quality, taking into account image sharpness (spatial resolution and vascular edge definition), contrast uniformity, motion robustness, artifact severity (e.g., ring artifacts), 3D reconstruction fidelity, and overall diagnostic confidence. Scores from the two independent readers were averaged to yield the final rating for each study.

### Diagnostic performance

CT diagnoses were independently reviewed by two radiologists with > 10 years of cardiovascular imaging experience, and discrepancies were resolved by consensus. For patients who underwent surgery, intraoperative findings were regarded as the definitive reference standard. In non-surgical cases, echocardiography served as the secondary reference standard. Correct diagnosis was defined as concordance between imaging and the reference standard, while misdiagnosis and missed diagnosis were defined as discordance or failure to detect relevant findings. These results were subsequently used to calculate diagnostic performance metrics (sensitivity, specificity, and accuracy) of CCTA in the evaluation of pediatric CHD.

### Statistical analysis

All statistical analyses were conducted using SPSS version 26.0 (IBM Corp., Armonk, NY). Continuous variables were expressed as mean ± standard deviation (SD) or median with interquartile range (IQR), while categorical data were presented as numbers and percentages. To compare quantitative data between the two groups (e.g., patient demographics, image noise, SNR, CNR, contrast agent dose, dose-length product, effective radiation dose, and subjective scores), a two-tailed Student’s t-test was used for normally distributed data with equal variances. Otherwise, the Mann-Whitney U test was applied. Interobserver agreement on image quality ratings was assessed using the intraclass correlation coefficient (ICC), with kappa values interpreted as follows: <0.4 indicating poor agreement, 0.4–0.75 indicating moderate agreement, and > 0.75 indicating good agreement. Diagnostic performance was compared using the non-parametric chi-square test. Statistical significance was defined as *P* < 0.05.

## Results

### Participant characteristics

A total of 72 pediatric patients with CHD (27 males, 45 females) were included in the study. There were no differences in gender, age, height, weight, and heart rate between the CE-Boost and CE-CT groups (all *P* > 0.05). Atrial septal defect (ASD) was the most common diagnosis, accounting for 60.4% in the CE-CT group and 68.3% in the CE-Boost group, followed by ventricular septal defect (VSD) (22.9% vs. 17.1%). Additional anomalies included the anomalous origin of the right coronary artery (AOCA) and partial anomalous pulmonary venous connection (PAPVC) in the CE-CT group. In addition, cases of partial atrioventricular septal defect (PAVS), aortic arch anomaly (DAA), and subaortic membrane (SAM) were seen in the CE-Boost group. Detailed demographic and clinical characteristics of the patients are presented in Table 1.

### Imaging protocol and radiation exposure

In terms of contrast agent usage (Table 2), the CE-CT group had an average contrast volume of 26.33 ± 7.57 mL, with an injection rate of 1.46 ± 0.39 mL/s; the CE-Boost group demonstrated a significant reduction in contrast usage, averaging 9.94 ± 3.39 mL (*P* < 0.001 vs. CE-CT group) while maintaining a comparable injection rate of 1.59 ± 0.48 mL/s. Although the difference in injection rate was not statistically significant (*P* = 0.251), the substantial reduction in contrast volume may help minimize patient risk of contrast media.

Regarding radiation dose, the dose-length product (DLP) in the CE-CT group was 20.05 (15.88–27.83) mGy·cm, with a CTDIvol of 1.25 (0.99–1.74) mGy, compared to 19.30 (14.58–21.40) mGy·cm and 1.21 (0.91–1.34) mGy, respectively, in the CE-Boost group. The differences between the two groups were not statistically significant (*P* > 0.1). The effective dose (ED) was 0.43 (0.30–0.59) mSv in the CE-CT group and 0.36 (0.34–0.40) mSv in the CE-Boost group, with the difference approaching statistical significance (*P* = 0.078). After adjusting for age using ANCOVA, the difference in effective dose (ED) between CE-CT and CE-Boost groups was not statistically significant (adjusted means: 0.52 vs. 0.41 mSv, *P* = 0.105) (Table S1). Age itself was not a significant covariate (*P* = 0.543), and the interaction between age and group was also non-significant (*P* = 0.977), supporting the validity of the ANCOVA model.

### Image quality analysis

The subjective imaging scores for the CE-CT and CE-Boost groups were 4.00 ± 0.95 and 4.10 ± 0.79, respectively, with no statistically significant difference between the two groups (*P* = 0.660). The inter-reader agreement, assessed using the ICC, was 0.712.

**Fig. 2:**
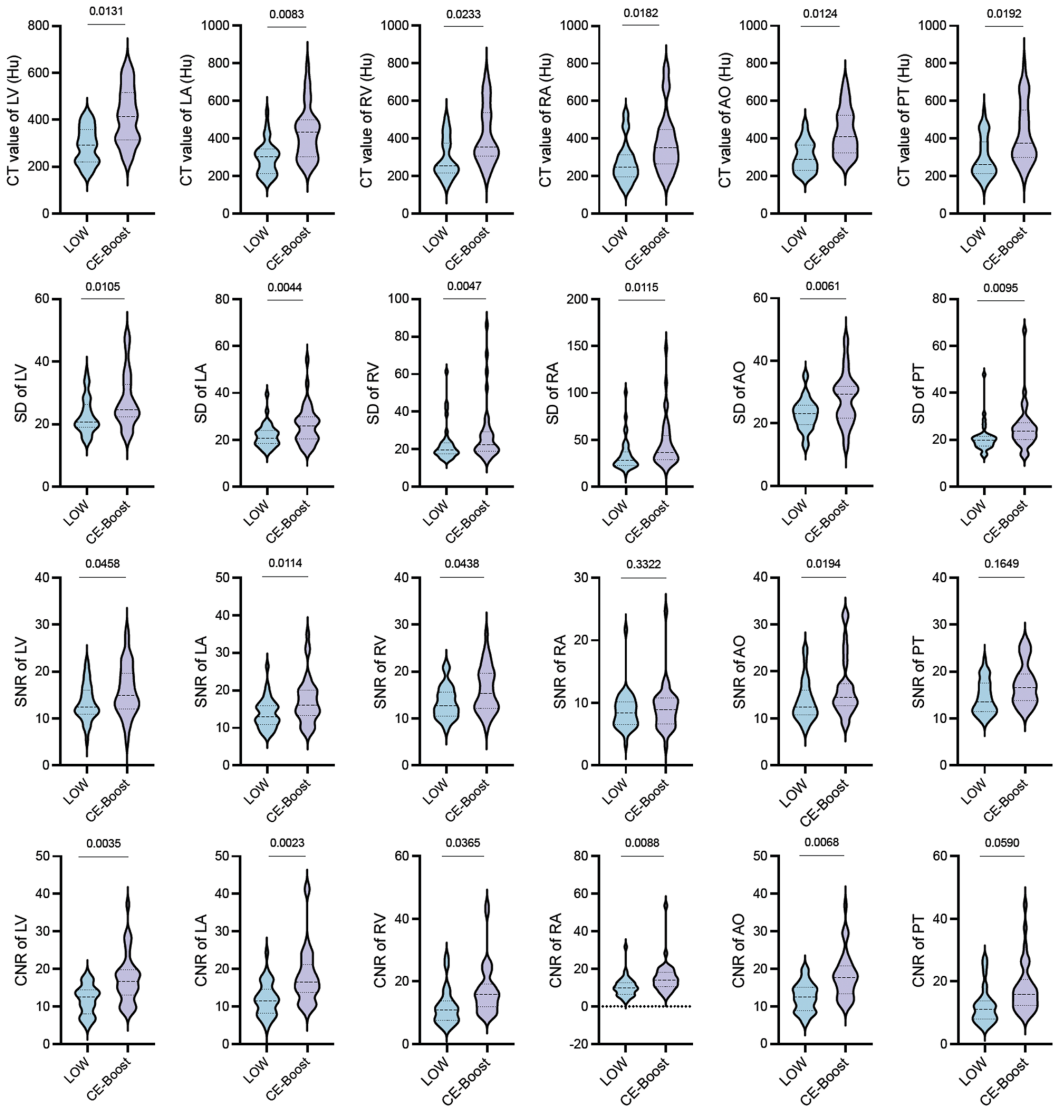
Objective evaluation of CE-Boost group in low-contrast initial and processed with CE-Boost images: a violin plot analysis. SD: standard deviation; CNR: contrast-to-noise ratio; SNR: signal-to-noise ratio; AO: aorta; PT: pulmonary trunk; LV: left ventricle; LA: left atrium; RV: right ventricle; RA: right atrium

In the CE-Boost group, the objective imaging scores between the initial low-contrast agent images and those processed with CE-Boost demonstrated statistically significant differences in CT attenuation values, SD, SNR, and CNR across all measured regions (all *P* < 0.05), except for SNR in the RV and PT, as well as CNR in the PT (Fig. 2).

The objective imaging scores for various cardiac structures, including the AO, PT, left ventricle (LV), left atrium (LA), right ventricle (RV), and right atrium (RA), showed no significant differences between CE-Boost imaging and conventional contrast-enhanced CT (CE-CT) (all *P* > 0.05). The comparison of CT attenuation values, SD, SNR, and CNR also revealed no statistically significant differences, with *P* > 0.05 (Table 3)​.

**Table 3:** Comparison of subjective and objective imaging scores between groups

Parameters	CE-CT group	CE-boost group	*P*-value
Subjective imaging scores	4.00 ± 0.95	4.10 ± 0.79	0.660
Objective imaging scores			
AO CT value (HU)	399.36 ± 94.54	432.82 ± 122.35	0.198
AO SD (HU)	26.70 ± 7.51	28.01 ± 8.21	0.316
AO SNR	15.80 ± 4.75	16.56 ± 6.43	0.437
AO CNR	18.16 ± 6.19	18.14 ± 6.07	0.985
PT CT value (HU)	364.34 ± 105.98	427.78 ± 159.17	0.191
PT SD (HU)	23.62 ± 5.47	25.01 ± 9.34	0.817
PT SNR	15.87 ± 4.94	17.40 ± 4.49	0.173
PT CNR	16.14 ± 6.66	17.75 ± 7.99	0.489
LV CT value (HU)	382.89 ± 88.82	415.59 ± 118.64	0.190
LV SD (HU)	24.37 ± 7.72	27.69 ± 8.91	0.086
LV SNR	16.25 ± 3.57	16.01 ± 5.40	0.824
LV CNR	17.23 ± 5.85	17.41 ± 6.52	0.899
LA CT value (HU)	378.13 ± 101.02	428.29 ± 138.84	0.131
LA SD (HU)	25.52 ± 8.00	26.26 ± 8.17	0.585
LA SNR	15.36 ± 3.64	17.15 ± 6.12	0.137
LA CNR	16.87 ± 6.08	17.95 ± 7.49	0.644
RV CT value (HU)	368.08 ± 110.19	414.47 ± 151.99	0.300
RV SD (HU)	24.66 ± 8.02	28.03 ± 15.86	0.919
RV SNR	15.77 ± 5.48	16.11 ± 4.89	0.782
RV CNR	16.31 ± 6.76	17.18 ± 8.16	0.835
RA CT value (HU)	343.59 ± 117.61	382.33 ± 161.38	0.248
RA SD (HU)	49.94 ± 29.43	46.25 ± 26.86	0.719
RA SNR	8.28 ± 3.71	9.17 ± 3.58	0.220
RA CNR	14.89 ± 6.90	15.25 ± 8.25	0.964

Data are presented as mean ± standard deviation. SD: standard deviation; SNR: signal-to-noise ratio; CNR: contrast-to-noise ratio; AO: aorta; PT: pulmonary trunk; LV: left ventricle; LA: left atrium; RV: right ventricle; RA: right atrium

### Diagnostic efficacy

**Fig. 3:**
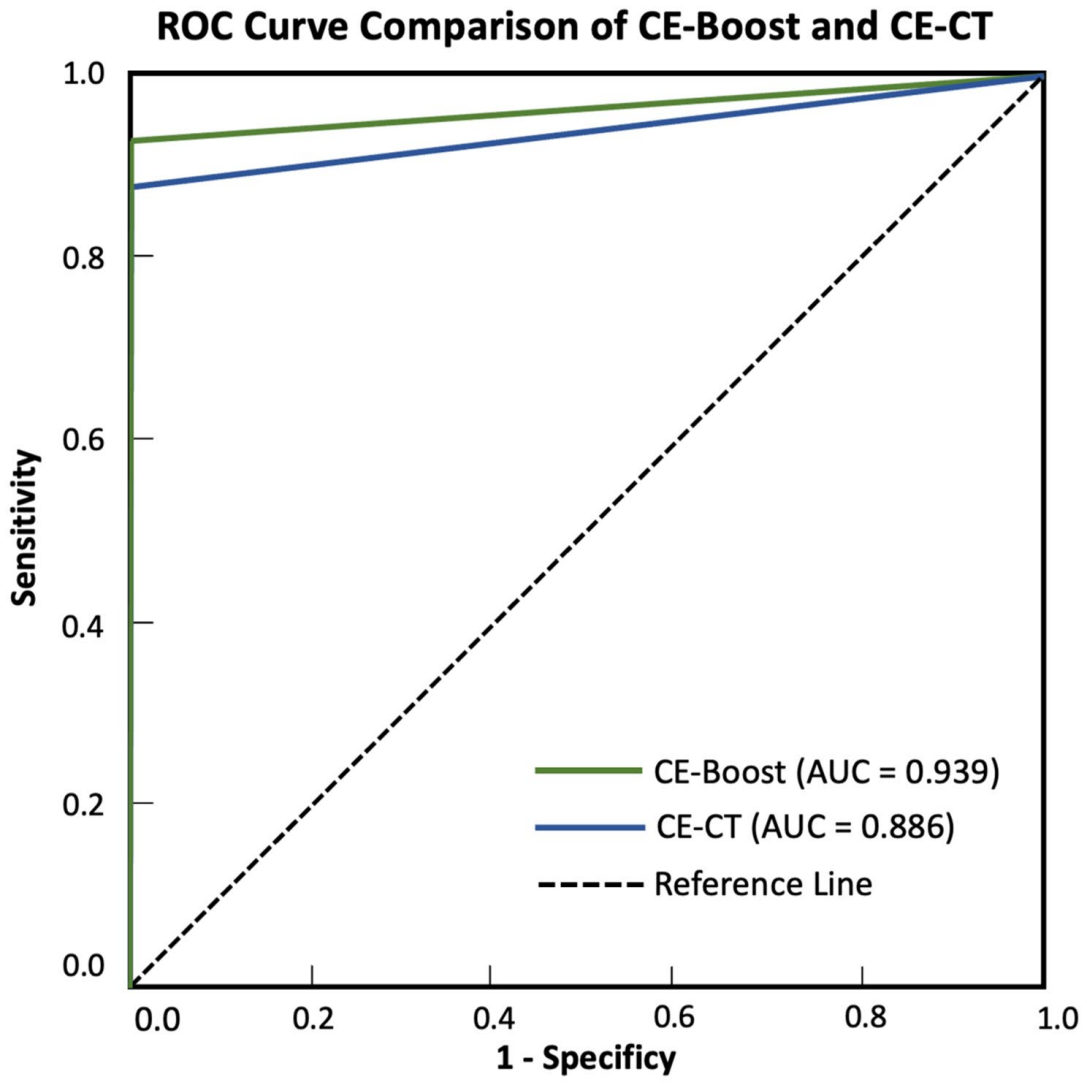
ROC Curve Comparison of CE-Boost and CE-CT. The green line represents CE-Boost with an AUC of 0.939; the blue line represents CE-CT with an AUC of 0.886

The diagnostic performance of CE-CT showed an accuracy of 88.54%, sensitivity of 89.36%, specificity of 79.59%, positive predictive value (PPV) of 79.17%, and negative predictive value (NPV) of 81.25%. In contrast, CE-boost demonstrated improvements across all metrics, with an accuracy of 93.90%, sensitivity of 92.86%, specificity of 87.76%, PPV of 87.50%, and NPV of 89.58%. Although CE-boost outperformed CE-CT, the p-value of 0.717 suggests that the difference between the two techniques was not statistically significant (Table 4). The ROC curve comparison between CE-Boost and CE-CT (Fig. 3) shows that CE-Boost has an AUC of 0.939, while CE-CT has an AUC of 0.886. The AUC of CE-Boost is slightly higher than that of CE-CT by 0.053.

**Table 4:** Diagnostic performance comparison of CE-CT and CE-Boost techniques

Group	Accuracy (%)	Sensitivity (%)	Specificity (%)	PPV (%)	NPV (%)	*P*-value
CE-CT	88.54%	89.36%	79.59%	79.17%	81.25%	0.717
CE-boost	93.90%	92.86%	87.76%	87.50%	89.58%	

PPV: positive predictive value; NPV: negative predictive value

## Discussion

This study evaluated the feasibility of using contrast enhancement boost (CE-Boost) combined with super-resolution deep learning reconstruction (SR-DLR) to reduce contrast agent dosage in pediatric patients with congenital heart disease (CHD). The key findings of this study are: (1) the contrast dose in the CE-Boost group was significantly lower than in the conventional CE-CT group; (2) CE-Boost maintained image quality comparable to CE-CT; (3) its diagnostic performance was equivalent to CE-CT. To our knowledge, this is the first study evaluating the efficacy of CE-Boost combined with SR-DLR in reducing contrast doses in pediatric patients.

Young children are more sensitive to contrast agents and radiation than adults, and their rapid heart rates and limited cooperation during scanning pose additional challenges for CTA [22]. This sensitivity requires careful consideration in imaging protocols to minimize risks while ensuring diagnostic accuracy, and a technique that minimizes contrast and radiation exposure is essential. In our cohort, all children underwent scanning under standard sedation protocols and free-breathing conditions. With the aid of SR-DLR, which ensured optimal spatial resolution [21], both non-contrast and contrast-enhanced acquisitions were successfully completed with minimal motion. Hydration before and after contrast administration to protect kidney function, use of iso-osmolar or low-osmolar contrast agents, which have a lower risk of nephrotoxicity, and lower effective contrast dose are some common methods to reduce contrast-related risks [23]. In pediatric and general radiology, image reconstruction algorithms and iterative reconstruction techniques are crucial in reducing noise while maintaining or enhancing the visibility of contrast-enhanced structures [24]. This is especially important in low-dose CT and MRI imaging, where reducing radiation exposure and optimizing contrast resolution is critical [24]. In our cohort, SR-DLR with CE-Boost was applied. CE-Boost increases iodine conspicuity and improves lesion-to-background contrast; functionally analogous capabilities are available on other CT platforms, including dual-energy/spectral CT iodine density maps and low-keV virtual monoenergetic imaging. SR-DLR is an advanced image reconstruction technique that utilizes deep convolutional neural networks trained with ultra-high-resolution CT (UHR-CT) images to enhance spatial resolution and reduce noise in CT imaging [25]. This technology has shown significant promise in improving image quality across various clinical applications, including coronary CT angiography and abdominal imaging [26].

In this study, pediatric CHD patients were divided into the low-contrast-dose group (CE-Boost group, injection of 0.5 mL per kilogram of body weight, *n* = 36) or the standard-scan group (CE-CT group, 1.5 mL per kilogram of body weight, *n* = 36), both groups undergoing imaging with SR-DLR. The contrast dose for CE-Boost (0.5 mL/kg) was lower than previously reported values, including 1–2 mL/kg in [27] (median age: 64 days) and 1.5 mL/kg in the photon-counting CT study by Timm Dirrichs et al. (median age: 66 days) [28]. Additionally, our study achieved higher SNR and CNR (CT attenuation: 18.0 vs. 62.0; SNR: 16.2 vs. 46.3; CNR: 18.0 vs. 62.0). Radiation doses for CE-Boost (0.36 mSv) and CE-CT (0.43 mSv) were also lower than those of photon-counting CT (0.50 mSv) and DSCT (0.52 mSv). These findings indicate that CE-Boost achieves a 33% reduction in contrast dose in patients of similar age while ensuring low radiation exposure and high diagnostic quality, supporting its role as a safer imaging alternative for pediatric CTA.

**Fig. 4:**
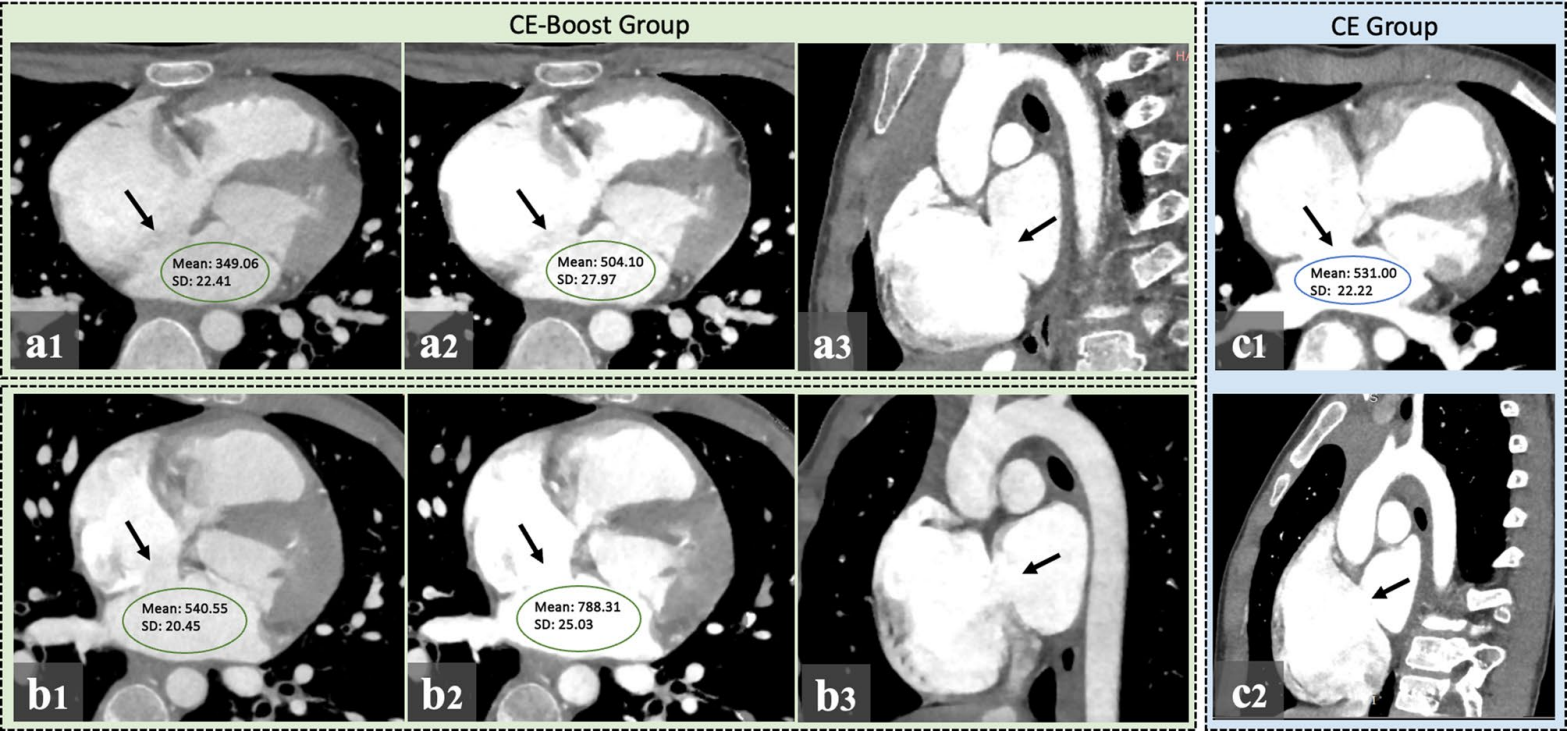
All images are of children diagnosed with ASD (atrial septal defect) (black arrow) and processed using SR-DLR, with a thickness of 0.5 mm. (**a**) and (**b**) are from children in the CE-Boost group; (**c**) is from a child in the conventional contrast agent group. (a) Weight 16.5 kg, 8.25 mL contrast agent. (a1) is the initial image, while (a2) and (a3) are reconstructed with CE-Boost. After CE-Boost processing, the average CT value of the left atrium increased by 155.04 compared to the unprocessed image (WW = 900, WL = 80). (b) Weight 20 kg, 10 mL contrast agent. (b1) is the initial image, (b2) and (b3) are reconstructed with CE-Boost. After CE-Boost processing, the average CT value of the left atrium increased by 247.76 compared to the unprocessed image. (WW = 1200, WL = 50) (c) Weighing 26 kg, 30 ml contrast agent, with a left atrium CT value of 531.00 (WW = 600, WL = 80)

We also evaluated the utility of CE-Boost in low-contrast cardiac CT imaging and investigated its performance under different imaging conditions. Our results demonstrate that in the CE-Boost group, images processed with CE-Boost showed statistically significant improvements (*P* < 0.05) in CT values, SNR, and CNR across all measured regions, except for the SNR of the RV and PT and the CNR of the PT (Fig. 4). When compared with conventional CE-CT, the CE-Boost protocol used only one-third of the contrast volume while maintaining the same contrast concentration and injection rate, thus achieving a comparable iodine delivery rate and minimizing potential timing mismatch in the time–attenuation curve (TIC) despite the narrower bolus width. The comparable intraluminal enhancement between groups therefore reflects genuine signal stacking from the CE-Boost reconstruction rather than off-peak acquisition. The significant differences in attenuation values between baseline and CE-Boost–processed images represent a true enhancement of iodine-related contrast rather than artificial modification of tissue HU, confirming the method’s ability to improve cardiac visualization in low-contrast scans. Moreover, compared with high-concentration CE-CT, CE-Boost substantially reduced contrast use (by 62.3%, *P* < 0.001) and decreased the incidence of artifacts such as beam-hardening, contrast pooling, and streak artifacts from nonuniform flow, thereby improving image uniformity and diagnostic confidence. This approach enables high-quality imaging with markedly lower contrast exposure—particularly valuable for pediatric patients and those with renal impairment—offering a safer and more sustainable option for longitudinal cardiovascular follow-up and clinical decision-making.

**Fig. 5:**
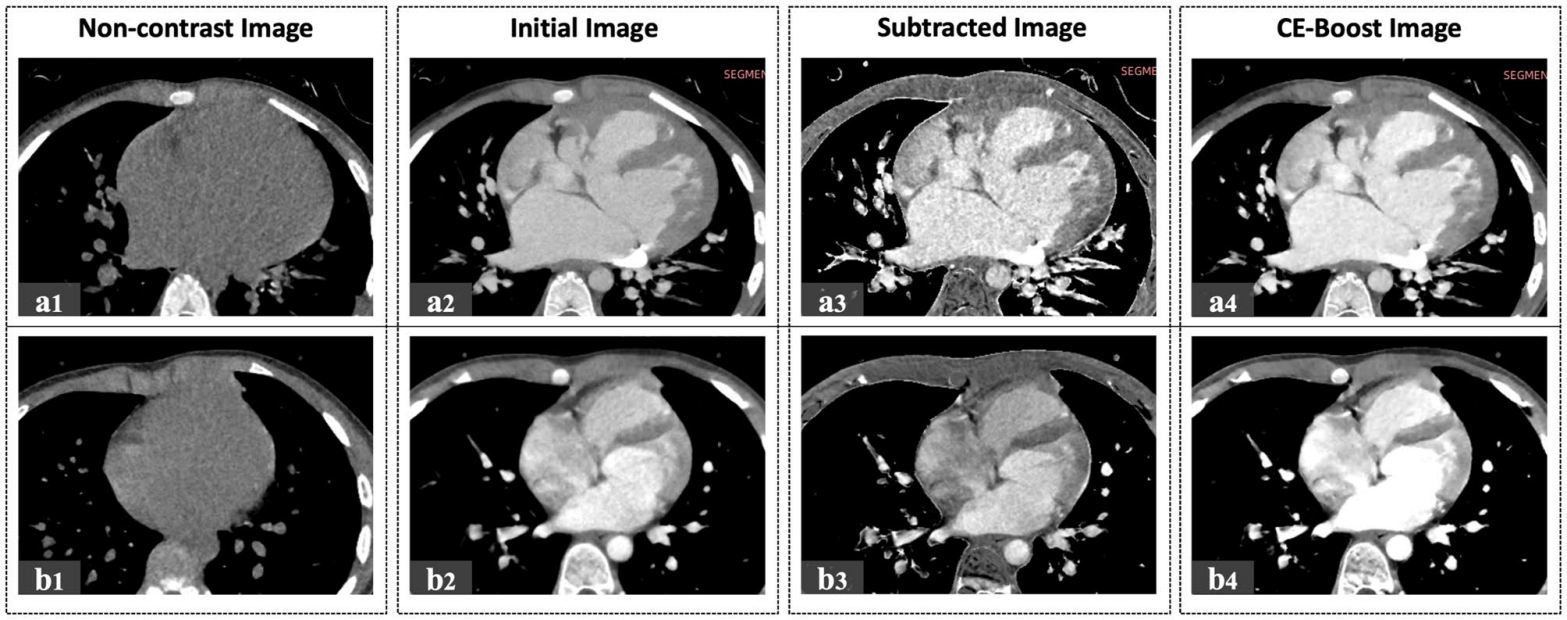
All images above are from the CE-Boost group with a thickness of 0.5 mm. (**a**) Weight 16 kg, 8 mL contrast agent. (**b**) Weight 15 kg, 7.5 mL contrast agent. a1, b1: the non-contrast calcium scoring scan. a2, b2: the initial contrast-enhanced image. a3, b3: the subtraction silhouette image obtained by subtracting the non-contrast scan from the contrast-enhanced image. a4, b4: CE-Boost post-processed images

The unique motion characteristics of the heart necessitate a distinct imaging approach for CE-Boost compared to other anatomical regions. As CE-Boost requires both non-contrast and contrast-enhanced acquisitions, the radiation dose from the non-contrast CT was included in the total dose calculation when comparing CE-Boost with conventional CE-CT. For the non-contrast phase, a calcium scoring protocol was applied to minimize radiation exposure, as it uses lower tube current and a restricted scan range (Fig. 5). Although calcium scoring is not routinely performed in pediatric patients, in our study it served solely as a low-dose acquisition method for subtraction, thereby ensuring registration accuracy while keeping radiation dose within the same range as conventional CE-CT protocols.

Based on our experience, when applying CE-Boost in low-contrast cardiac CT, it is crucial to use the Target technique during the non-contrast scan, aligning acquisition timing with 35%-55% of the cardiac cycle (end-systole). This ensures synchronization with the contrast-enhanced phase, improving image registration accuracy.

In wide-detector CT, HIR, commonly used in clinical practice, only incorporates statistical system modeling and forward projection steps. When CE-Boost is applied to routine pediatric scans, there is an increased risk of image noise. To address this, we incorporated SR-DLR to enhance spatial resolution and reduce noise [29]. Ye et al. ^30^ further confirmed that an advanced reconstruction approach, such as model-based iterative reconstruction, effectively mitigates CE-Boost-related noise. The combination of CE-Boost and SR-DLR is critical in detecting subtle congenital heart lesions and delineating small cardiac structures in 3D or virtual reality models, enhancing diagnostic precision and clinical utility [28].

CE-Boost enhances iodine signal intensity by integrating iodine distribution maps with contrast-enhanced images, improving overall image quality and enhancing visualization of poorly opacified vascular branches, peripheral arteries, and veins [31]. Previous studies have demonstrated its effectiveness in optimizing angiographic imaging and perfusion accuracy in various vascular territories, including the carotid and vertebral arteries [32], aortic aneurysms [33], pulmonary arteries [30], and the portal venous system [34]. However, its application in reducing contrast media usage has not been extensively studied. Our study, conducted in pediatric congenital heart disease—a population characterized by poor compliance, high heart rates, and complex hemodynamics—demonstrates the feasibility of combining CE-Boost with SR-DLR. This approach holds promise for broader applications, including ultra-low contrast dose imaging in infants, adult cardiovascular disease assessment, oncologic imaging, and safe imaging for patients with chronic kidney disease. By reducing contrast media requirements without compromising image quality, the integration of CE-Boost and SR-DLR enhances diagnostic accuracy and mitigates contrast-related risks, promoting safer and more efficient imaging practices.

This study has certain limitations. First, the cohort consisted primarily of older pediatric patients, with a limited number of neonates, potentially restricting the generalizability of our findings across all age groups. Additionally, the relatively small sample size resulted in a limited representation of congenital heart disease subtypes, which may introduce a degree of statistical bias. The window width and level during MPR-based diagnosis and scoring were adjusted individually by each radiologist according to their clinical experience. While not standardized, this approach reflects real-world practice and was mitigated by the expertise of both readers (over 10 years each). Although injection parameters were standardized, hemodynamic heterogeneity among CHD subtypes cannot be excluded. Physiologic proxies of cardiac output were not systematically collected, and TIC data were unavailable, preventing direct confirmation of enhancement uniformity. These factors may have introduced residual bias and warrant further validation in prospective studies with comprehensive hemodynamic assessment.

## Conclusion

This study demonstrates that CE-Boost can maintain high-quality imaging while significantly reducing contrast agent usage without substantially increasing radiation doses. When combined with SR-DLR, this technique further enhances imaging safety and diagnostic value, making it particularly suitable for CT evaluation in CHD patients. Additionally, it may serve as a viable alternative for low-contrast imaging in select patient populations, supporting more precise clinical interventions and diagnoses.

## Supplementary Information

Below is the link to the electronic supplementary material.

Supplementary Material 1

Supplementary Material 2

## Data Availability

The datasets used and/or analyzed during the current study are available from the corresponding author upon reasonable request.

## Abbreviations

CCTA: Cardiac computed tomography angiography
CHD: Congenital heart disease
SR-DLR: Super-resolution deep learning reconstruction
PIQE: Precise IQ engine
SD: Standard deviation
CNR: Contrast-to-noise ratio
SNR: Signal-to-noise ratio
DLP: Dose-length product
CTDIvol: The volume-weighted CT dose index
ED: Effective dose
ICC: Intraclass correlation coefficient
AO: Aorta
PT: Pulmonary trunk
LV: Left ventricle
LA: Left atrium
RV: Right ventricle
RA: Right atrium
ASD: Atrial septal defect
VSD: Ventricular septal defect
PDA: Patent ductus arteriosus
AUC: Area under the curve
