# Supplementary material for: Impact of contrast enhancement boost and super-resolution deep learning reconstruction on pediatric congenital heart disease CTA scans: ultra-low contrast dose
Source: BMC Med Imaging. 2025 Nov 18;25:477. doi: 10.1186/s12880-025-02015-2 (PMC12625121; doi:10.1186/s12880-025-02015-2)
Supplement: Supplementary file 2 — Supplementary Material 2 [file 12880_2025_2015_MOESM2_ESM.docx]

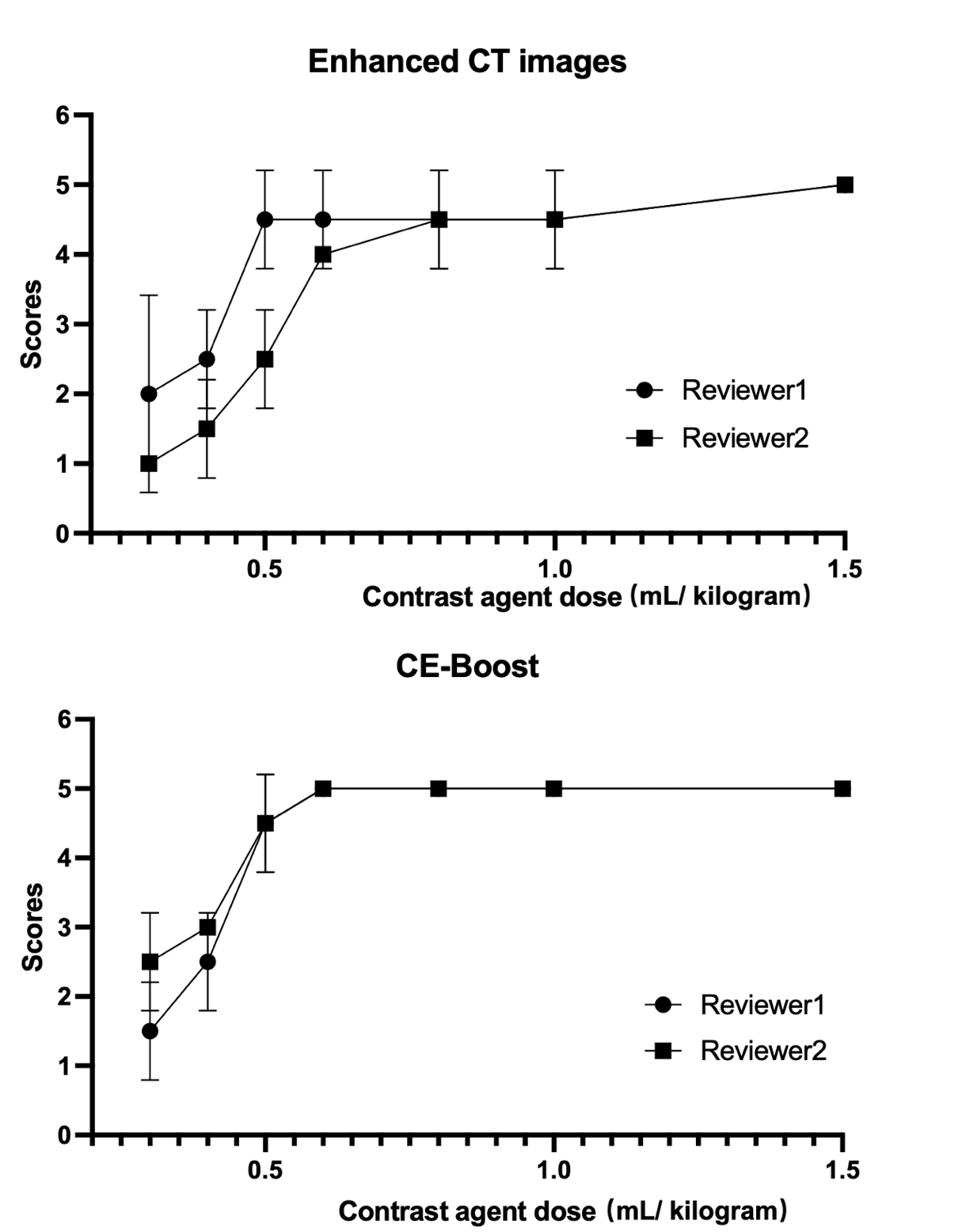


**Supplemental Figure S1. Subjective image quality scores across varying contrast agent doses in pre-study testing.** Image quality was rated by two independent senior radiologists (Reviewer 1 and Reviewer 2, each with >10 years of experience) using a 5-point Likert scale (1 = non-diagnostic, 5 = excellent). The upper panel shows scores for conventional enhanced CT images, while the lower panel shows scores for CE-Boost images. For each dose level (0.3–1.5 mL/kg), two pediatric patients were scanned, and the average score ± standard deviation is shown. In CE-Boost images, diagnostic scores plateaued at 0.5 mL/kg, suggesting this dose achieves optimal image quality with minimal further gain from increased contrast volume.
